# The Prophylactic Uses of Chemotherapy
*A paper read before the Bristol Medico-Chirurgical Society on Wednesday, 9th March, 1949.


**Published:** 1949-10

**Authors:** J. A. Boycott

**Affiliations:** Director, Public Health Laboratory Service, Taunton


### THE PROPHYLACTIC USES OF CHEMOTHERAPY*

BY

J. A. Boycott, D.M.,

*Director, Public Health Laboratory Service, Taunton.*

---

The use of drugs for the prevention as opposed to the cure of infectious disease has a long history, but for a century quinine as a prophylactic of malaria was probably the only example of such use whose efficacy would stand critical examination. Listerian practice introduced the local use of antiseptics, but except for the general agreement that this was an improvement in surgical procedure there is little statistical information which allows of comparison with other methods of preventing the infection of surgical wounds. Since the introduction of the sulphonamide drugs and penicillin the bacteriostatic efficiency of these has encouraged their prophylactic use in a variety of conditions. With a few exceptions the trials have been sporadic and incompletely controlled, but some general conclusions may be drawn from the available evidence. What follows is applicable to these drugs only. Irrespective of chemical composition, they share the properties of very slight toxicity, bacteriostatic action in low concentrations, and, under certain conditions, the power of inducing resistance to their action in the bacteria whose invasion they are intended to combat. This last is shared with many antiseptics, and that it has attained some notoriety for these drugs is, in part at any rate, a consequence of their other desirable qualities which allow their use for long periods without apparent danger or discomfort to the patient.

The dangers in the development of drug resistance have been best illustrated in the large-scale experiment conducted by the U.S. naval authorities during the last war.[1, 2] Streptococcal infections of the upper respiratory tract in barracks were sufficiently numerous to embarrass the training programme. Five training depots were divided into subject and control groups, and each member of the former received 1.0 gramme sulphadiazine daily. The effect was seen at once in a dramatic fall in the morbidity rates for scarlatina and rheumatic fever. Toxic effects were seen in 1 per 10,000 of those treated. Further inquiry showed that 0.5 gramme was equally effective : and, on the strength of these findings, this daily dose was

* A paper read before the Bristol Medico-Chirurgical Society on Wednesday, 9th March, 1949.

administered to the quarter of a million men in training. Within a few months it was noticed that morbidity rates for streptococcal infections had risen above the initial levels and that cases of considerable severity were common. The experiment was abandoned in the majority of establishments, and in these the morbidity rates fell. They remained high in those where prophylaxis was continued and were not influenced by an increase of the daily dose to 1.5 grammes. At this point the experiment was ended.

A detailed bacteriological survey had shown that the recrudescence of infection after the initial fall was due to three serological types of hæmolytic streptococcus, 3, 17, and 19, all markedly invasive and resistant to sulphadiazine. (An incidental result of the experiment was the dissemination of these types throughout the U.S. Navy, outside the establishments where prophylaxis had been used.) Drug resistence may be induced *in vitro* and *in vivo* in many sensitive bacteria by prolonged exposure to sublethal doses of bacteriostatic substances, but there is also evidence to show that a small proportion of normally sensitive bacteria are, in fact, resistant, and under the same conditions will in the end outgrow and replace the sensitive strains. Serial throat swabs from healthy children treated with bacteriostatic substances show a replacement of the sensitive by a resistant flora ; withdrawal of the drug is followed by a more or less complete retuın to the status *quo ante.*[3] By the time the results of the experiment in mass prophylaxis had been appreciated it was too late to discover which mechanism had been responsible for the prevalence of the resistant types. The conclusion, however, is clear ; that long-continued chemo-prophylaxis is a dangerous proceeding. Whether there are greater risks which justify its use is open to argument. There is evidence that recurrences of rheumatic fever are associated with streptococcal throat infections, and it is probable that these may be prevented by a small daily dose of sulphanilamide.[4] Whether such a regimen is justified must obviously depend on the degree of cardiac damage existing and the risk of further infections. Since such cases must be kept in hospital for some months it is probable that freedom from infection might be gained more safely by the strict bacteriological control of new patients and nursing staff. At the same time the production of a drug-fast strain among a stationary hospital population involves far less risk to that population and to the outside world than it would in an ever-changing community such as was involved in the American experiment.

The benefits of short-term prophylaxis are best shown in the prevention of meningococcal infections. During the war this was employed successfully in many instances among the armed forces, although few such essays were described and fewer satisfactorily controlled. Kuhn and others,[5] however, showed that 4 grammes of sulphadiazine spread over two days reduced both infection and carrier rates to a low level. But once again it should be emphasized that to be effective such treatment must be applied to all the population at risk. No benefit is likely to result if during an epidemic such treatment is given to the patients in one general practice: since, as soon as the sterilizing effect is ended—perhaps a few days—they would be exposed to re-infection from carriers outside the practice. But in the boarding-school or other closed community it is both valuable and apparently free from risk.

We have made some attempts to apply similar measures to streptococcal infections in boarding-schools. Failure in these attempts seems to be the common experience and explained most easily by the relative resistance to treatment of streptococcal infections.[6, 7] To bring an epidemic to an end it is necessary to sterilize not only those suffering from clinical infections but also carriers. To free the latter of streptococcal infection is not always easy and failure involves a risk of producing a drug-resistant strain. Dosage must, therefore, be heavy, and it is to too small a dosage that many of our failures must be attributed. By the time assistance is sought the epidemic strain of streptococcus is usually widespread in the community, and the most promising method of ending an epidemic in our experience is the search of the entire population at risk for carriers and their individual vigorous treatment. Throat carriers may be treated while ambulant, but nasal carriers involve a greater risk[8] and should be isolated until free from infection. A more hopeful prospect of the prevention of streptococcal infections is seen in the use of sulphadiazine or penicillin to ward off the complications of scarlatina or measles in an isolation ward. In spite of some unsuccessful trials it seems to be generally agreed that there is value in such methods, so long as they are limited to periods when a highly invasive streptococcus is in circulation and when their protective value is backed up by sanitary measures to prevent cross-infection. In such circumstances, too, carriers must be detected as a matter of course.

Attempts at the prophylaxis of staphylococcal infections have not been promising. A large proportion of the population harbour pathogenic staphylococci in their noses, and moderate prophylactic doses of, say, penicillin will tend rather to produce resistant strains than to end the carrier state. The dramatic success attained in meningococcal infections appears to be due to the far greater susceptibility of the Neisseria which allows the carrier to be sterilized quickly and surely. Individual prophylaxis of gonorrhœa with sulphadiazine was attempted during the war with some success; all men returning to camp received treatment lasting until the next day and infections both with gonorrhœa and soft sore decreased in comparison with those in control groups.[9] The numerous current strains of N. gonorrhϾ resistant to sulphadiazine suggest that success with such methods to-day would be doubtful, and recent reports of similar attempts using penicillin have greater promise.[10] It is doubtful whether a penicillin-resistant strain of N. gonorrhœæ has yet been found.

The prophylactic uses of chemotherapy as an aid to surgery range from the drop of iodine on the small boy's knee to the " blanket " cover of major operations involving risks of generalized infection. The efficacy of the local application of an antiseptic to a superficial wound lends itself to experimental investigation. Recent work using mice shows that sulphanilamide powder is the best preventive of streptococcal infections while dyes, *e.g.* " blue paint ", are scarcely less successful, and, since they are effective against a wider range of organisms, probably the application of choice.[11] Among many preparations tested " tincture of iodine " came a bad last. Once more this work underlines the view that no local application inhibits infection in a deep wound. The delay between wounding and adequate surgical treatment which is inherent in warfare was a great stimulus to the use of chemoprophylaxis. At the time there was small opportunity or enthusiasm for controlled experiment: and such varieties of wound and treatment were employed that it is difficult in retrospect to make dogmatic claims for the superiority of one method over another. Nevertheless, study of the fatality rates from all theatres of war suggests very strongly that while such treatment can seldom replace surgical repair, it is, provided it is used without parsimony, a most valuable adjuvant.

We have, in fact, reasonable grounds for assuming that chemoprophylaxis may be of value in medicine and surgery and that the same general rules apply in each field. To be effective it must be used in such doses as will prevent invasion of the body by pathogenic bacteria, and, in many instances, remove potentially pathogenic bacteria from the mucous surfaces. Because a patient has no clinical signs of infection is not a valid argument against treatment with massive doses under these circumstances. The major risk is the production of bacterial strains resistant to the action of the drug used. This is best avoided if treatment is limited to the shortest possible time effective in removing infection. Where risks of re-infection are high, *e.g.* the pupils in a day school or the surgical wound whose condition requires repeated exposure in improvised dressing stations, judgment must be used on the justification for continued prophylaxis. In the first case the answer is probably " no ", in the second " yes ". At the same time it must not be forgotten that almost every day another arrow is added to the doctor's quiver, and that should an organism be rendered resistant to one drug there are, in most instances, others which may be used with success. The American experiment described at the beginning of this paper threw doubt on the value of chemoprophylaxis. This is a pity since it has undoubted possibilities for good. It is a subject which presents few major difficulties to the experimental approach and should be pursued.

### REFERENCES

[1] Coburn, A. F. (1944), *J.A.M.A.*, **126,** 88.
[2] *J.A.M.A.* (1945), **129,** 921.
[3] Siegel, M. (1946), *Am. Jour. Hyg.*, **44,** 257.
[4] Barclay, P. E., and King-Lewis, F. L. (1945), *Lancet,* ii. 751.
[5] Kuhn, D. M., Nelson, C. T., Feldman, H. A., and Kuhn, L. R. (1943), *J.A.M.A.*, **123,** 335.
[6] Vollum, R. L., and Wilson, G. S. (1945), *B.M.J.*, i. 545.
[7] Hamburger, M., Jnr., and Lemon, H. M. (1946), *J.A.M.A.*, **130,** 836.
[8] Hamburger, M., Jnr., et al (1945), *J. Infect. Dis.*, **77,** 96.
[9] Loveless, J. A., and Denton, W. (1943), *J.A.M.A.*, **121,** 827.
[10] Eagle, H., et al (1948), *Pub. Hlth. Rep. Wash.*, **63,** 44.
[11] Gordon, J., et al (1947), *J. Hyg.*, **45,** 297.

